# Pheochromocytoma in a Patient With Chronic Hypotension: A Case Report

**DOI:** 10.7759/cureus.93600

**Published:** 2025-09-30

**Authors:** Meredith Harkema, Emily Brady, Maximo Llaudes, David Meir-Levi, Jacqueline Oxenberg

**Affiliations:** 1 Medicine, Philadelphia College of Osteopathic Medicine, Philadelphia, USA; 2 Anesthesiology, University of Scranton, Scranton, USA; 3 Pathology, Lehigh Valley Health Network, East Stroudsburg, USA; 4 Vascular and Endovascular Surgery, Lehigh Valley Health Network, East Stroudsburg, USA; 5 Surgical Oncology, Lehigh Valley Health Network, East Stroudsburg, USA

**Keywords:** adrenalectomy, metanephrines, normetanephrines, paraganglioma, pheochromocytoma

## Abstract

Pheochromocytomas are neuroendocrine tumors that commonly present with hypertension, headache, palpitations, perspiration, and facial flushing. The diagnosis is typically made with imaging demonstrating an adrenal mass and elevated metanephrines and normetanephrines. The recommended treatment is medical optimization and adrenalectomy. Here, we present the case of a patient referred to our surgical oncology clinic with diagnostic challenges due to chronic hypotension and only heart palpitations. She was found to have a peripherally located adrenal mass extending over the renal hilum with an otherwise normal adrenal gland. Elevated plasma and urine normetanephrines were found, so the patient underwent complete resection of the tumor with en bloc adrenalectomy and was ultimately found to have a pheochromocytoma.  After surgery, she has no evidence of disease and has had resolution of palpitations.

## Introduction

Pheochromocytomas are rare neuroendocrine tumors of the adrenal gland that cause hypersecretion of catecholamines causing a classic triad of symptoms, including headache, palpitations, and sweating, but also paroxysmal hypertension [1-3]. Diagnosis typically includes documentation of excess release of catecholamines and anatomical documentation of a tumor [3]. While the most common symptom is hypertension, either sustained or paroxysmal, a small portion of patients can be normotensive [1]. This hypertension is secondary to catecholamine hypersecretion and can result in significant cardiovascular morbidity. Therefore, surgical resection is typically recommended, preceded by at least seven days of alpha blockade to prevent dangerous perioperative and intraoperative hemodynamic instability [2,3]. 

Imaging helps determine the location and size of the tumor(s) and metastases and guides surgical planning. Imaging is also important to ensure lesions are unilateral and to determine extra-adrenal locations. According to the 2017 World Health Organization (WHO) classification, pheochromocytomas, defined as adrenal tumors, and paragangliomas, defined as extra-adrenal tumors, were only distinguished by location [3]. In the case of an extra-adrenal location, the tumor can be a functional paraganglioma, which is often located in the tubercle of Zuckerkandl. While either an adrenal protocol computed tomography (CT) or magnetic resonance imaging (MRI) of the abdomen and pelvis should be performed, functional imaging may also be helpful, particularly with large tumors or when there is suspicion for metastatic disease [2,4]. 

In this report, we present the workup and management of a woman with chronic hypotension and heart palpitations who was found to have a slow-growing retroperitoneal mass. While her serum and urine metanephrines were found to be elevated, the location on imaging and chronic hypotension created diagnostic and treatment challenges. Through the care of a multidisciplinary team, the patient was successfully able to undergo surgical resection of what was found to be a peripherally located left adrenal tumor, for which final pathology confirmed a pheochromocytoma.   

## Case presentation

**Figure 1:**
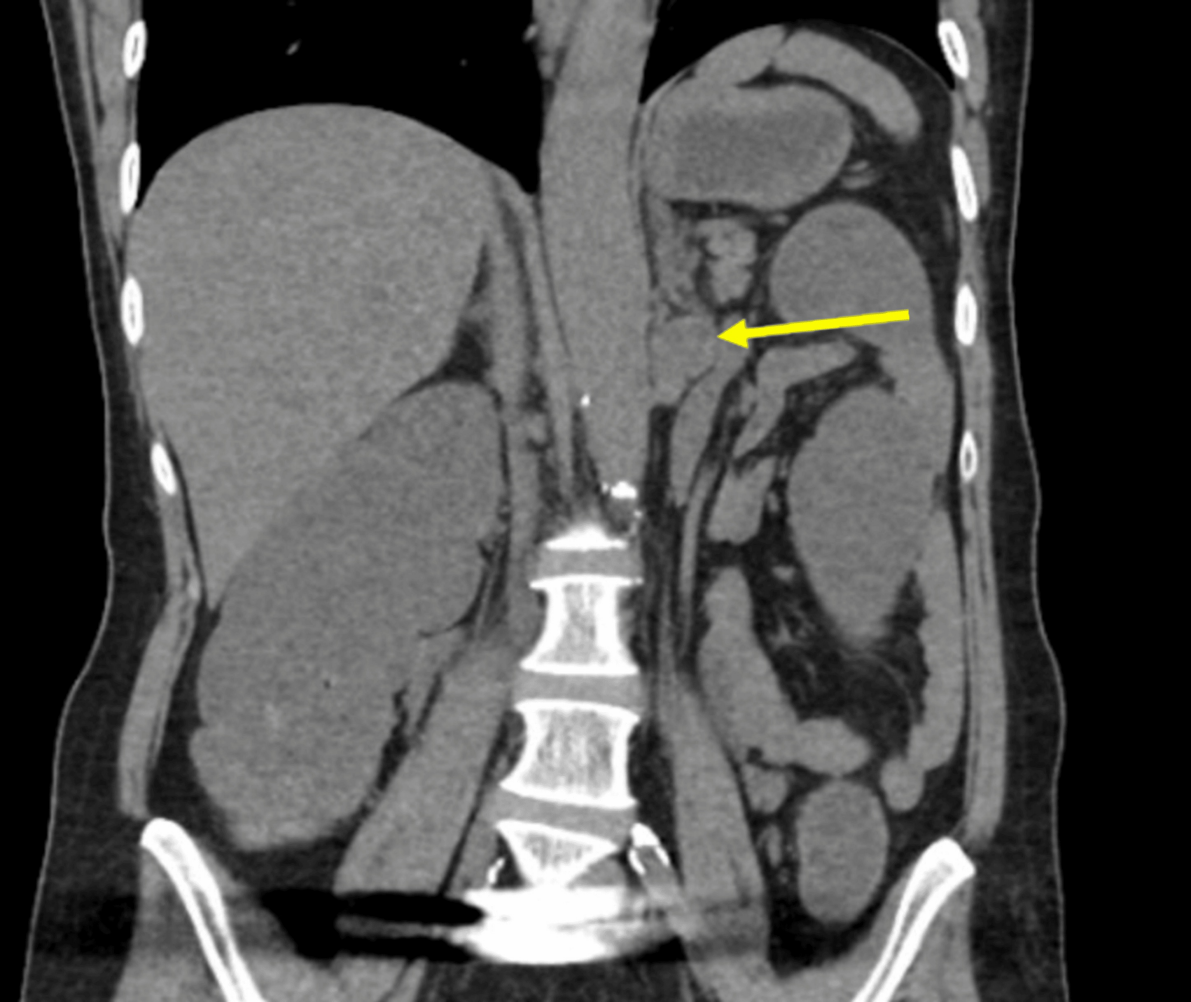
Retroperitoneal mass (yellow arrow) seen on non-contrast CT in September of 2021 CT: computed tomography

A 63-year-old woman was referred to our surgical oncology service at Lehigh Valley Hospital, East Stroudsburg, PA, USA, by her primary care provider for an incidental soft tissue mass in the left retroperitoneum, located between the adrenal gland and kidney, found on CT in May 2024 for abdominal pain. While prior imaging reports did not note the mass, direct review of a non-contrast CT showed the mass was present back in September of 2021, when it measured 2.2×1.9 cm (Figure 1). The patient's biggest concern at the initial evaluation was her palpitations, which were worse in the morning and associated with shortness of breath. She was also on 50 mcg/hour Duragesic patches, for which she admitted to symptoms of withdrawal when having problems with them sticking to her skin. While these issues were resolved when seen, narcotic withdrawal symptoms were also considered. Her symptoms were negative for headaches, abdominal pain, chest pain, facial flushing, and fluctuations in blood pressure.

Prior to referral to surgical oncology, plasma metanephrines and plasma free normetanephrines were also checked by her cardiologist, since she was experiencing heart palpitations. While plasma metanephrines were normal, free normetanephrines were greater than two times the upper limit of normal and therefore raised the concern for pheochromocytoma [5] (Table 1). However, her blood pressure was 95/45 mmHg, and the patient was on 5 mg midodrine daily for persistent hypotension. A decision was made to proceed with repeat biochemical workup, off of midodrine, to ensure no false elevation secondary to the medication [6]. She was also referred to cardiology for further workup of the palpitations, whereas the workup revealed no cardiac cause.  

**Table 1:** Biochemical workup of the retroperitoneal/adrenal mass on and off midodrine Cells with "-" indicate laboratory tests that were not initially investigated. CRT: creatinine ratio; DHEA: dehydroepiandrosterone

Test	On midodrine	Off midodrine	Reference range
Metanephrines, fractionated	0.31 nmol/L	0.23 nmol/L	0-0.49 nmol/L
Normetanephrines, free	11.43 nmol/L	7.88 nmol/L	0-0.89 nmol/L
Urine metanephrines (CRT)	-	159 ug/g	0-300 ug/g
24-hour urine metanephrines	-	16 ug/d	36-229 ug/d
Urine normetanephrines (CRT)	-	2809 ug/g	0-400 ug/g
24-hour urine normetanephrines	-	2949 ug/d	95-650 ug/d
Cortisol (dexamethasone suppression test)	-	0.6 ug/dL	<1.8 ug/dL
Adrenocorticotropic hormone	-	2.8 pg/mL	7.2-63.3 pg/mL
Aldosterone	-	7.5 ng/dL	<31 ng/dL
Aldosterone/renin ratio	-	12.4	≤25
DHEA	-	0.28 ng/mL	0.63-4.7 ng/mL

**Figure 2:**
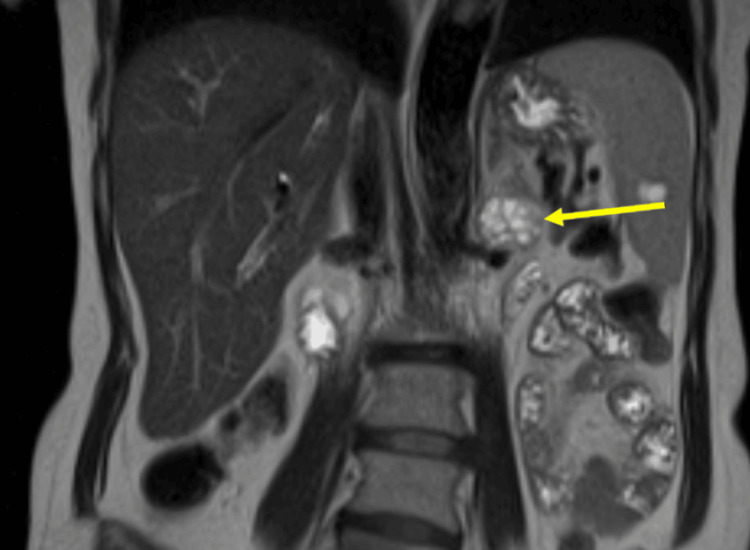
Retroperitoneal mass (yellow arrow) on MRI MRI: magnetic resonance imaging

**Figure 3:**
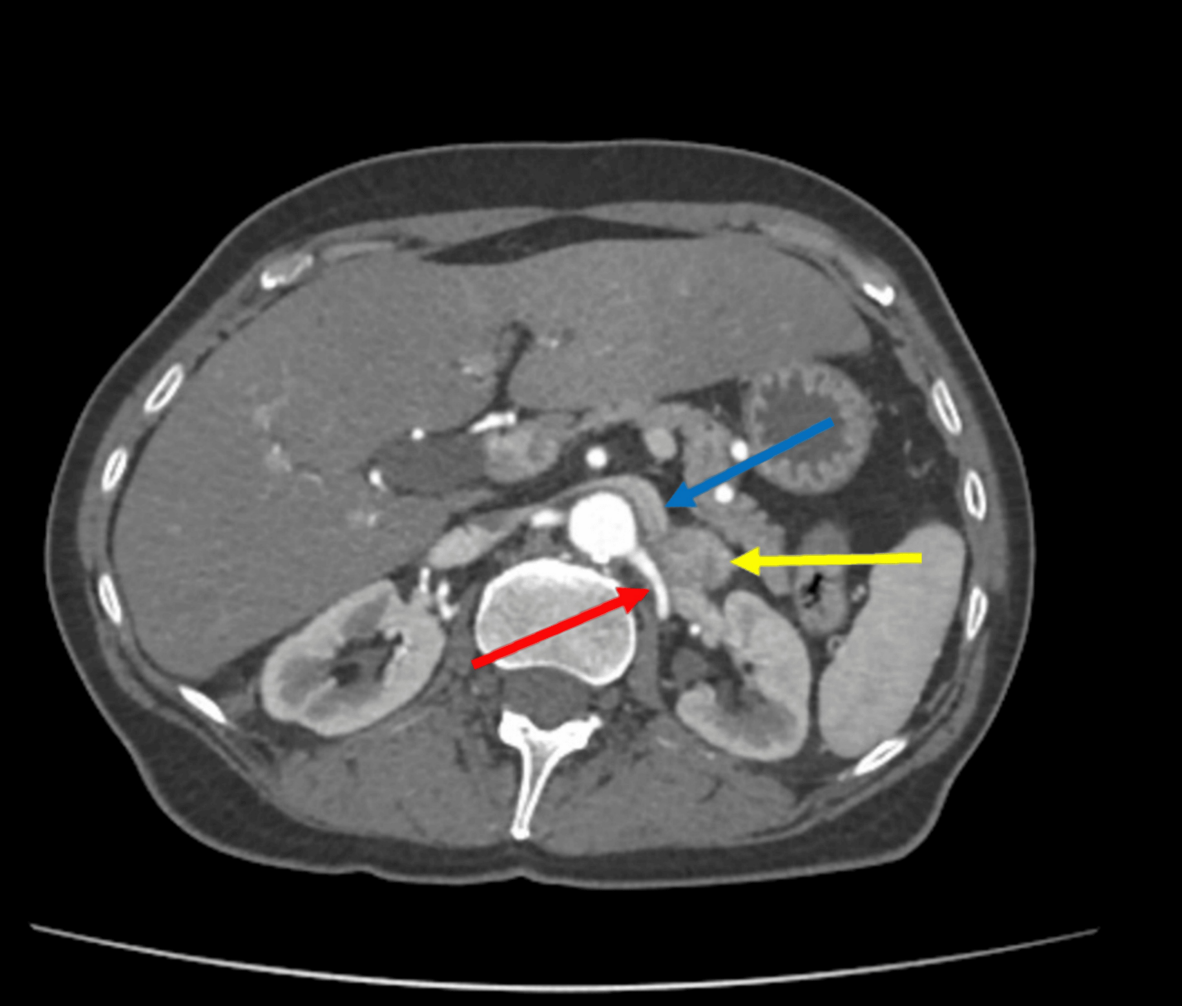
Cross-sectional CT imaging showing the retroperitoneal mass (yellow arrow) abutting the renal artery (red arrow) and displacing the renal vein (blue arrow) CT: computed tomography

**Figure 4:**
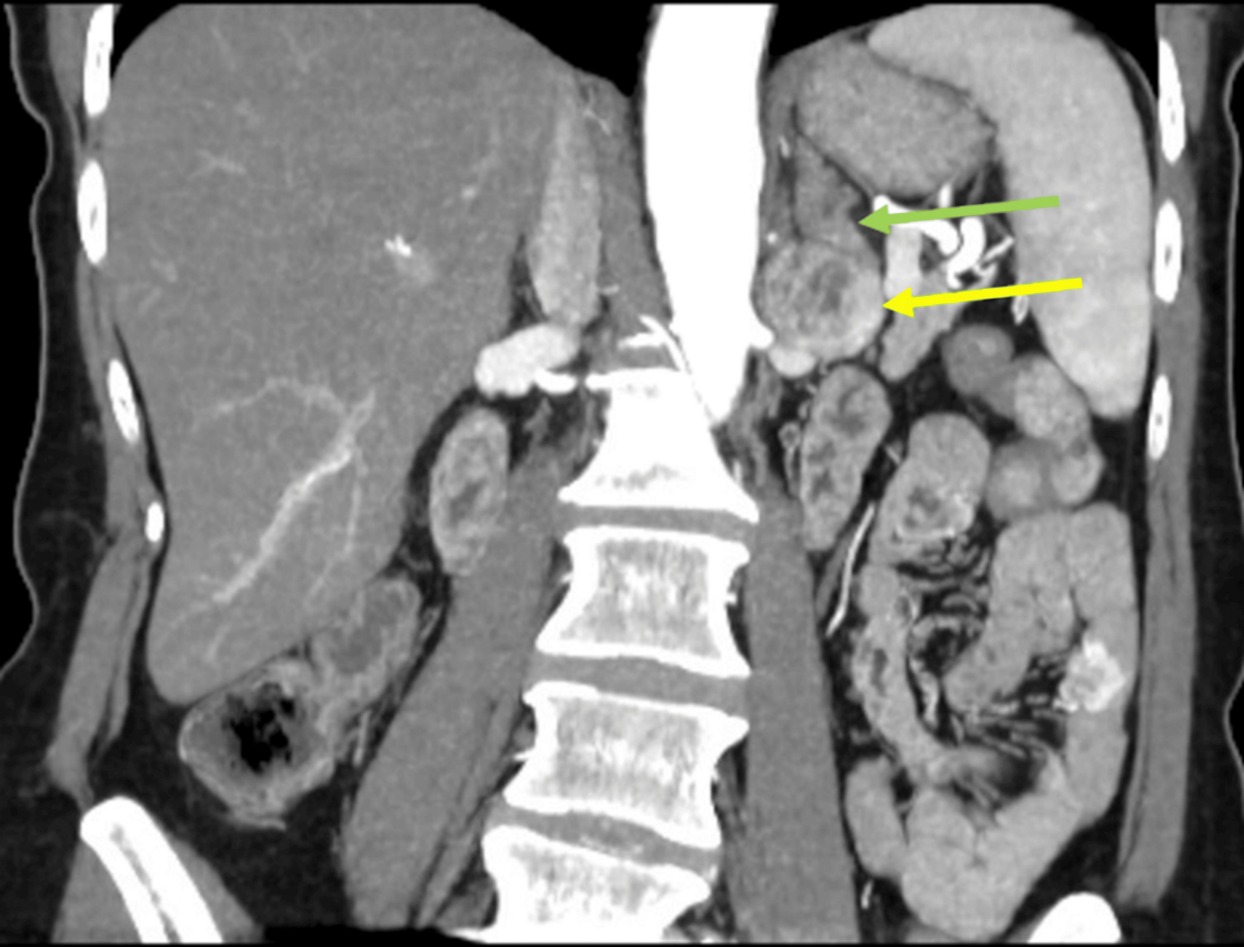
Retroperitoneal versus adrenal mass (yellow arrow) in comparison to otherwise normal adrenal gland (green arrow)

Since it was unclear if the mass was contiguous with the adrenal gland, paraganglioma or neurogenic tumor was also considered, and an MRI was ordered. The adrenal protocol MRI was performed in June 2024 and showed a 3 cm heterogeneously enhancing mass in the left retroperitoneum at the level of the renal hilum without a clear connection to adjacent organs, abutting the tip of the left adrenal gland (Figure 2). Repeat biochemical workup was also performed, including for a possible functional adrenal tumor. With midodrine held for two weeks, plasma and 24-hour urine metanephrines were still normal, and plasma and 24-hour urine normetanephrines were still elevated (Table 1). All other biochemical workup was negative for a different type of hyperfunctioning tumor. Due to the enlarging size of the mass and the labs demonstrating elevated normetanephrines, the decision was made to proceed with surgical resection of the mass and possible adrenal gland. Given the compression of the mass on the renal vein, a CT angiogram was ordered for surgical planning, which showed the mass to be in close contact with the left renal artery with a fat plane maintained, and the left renal vein was displaced (Figures 3-4). The adrenal vein could not be clearly identified.   

**Figure 5:**
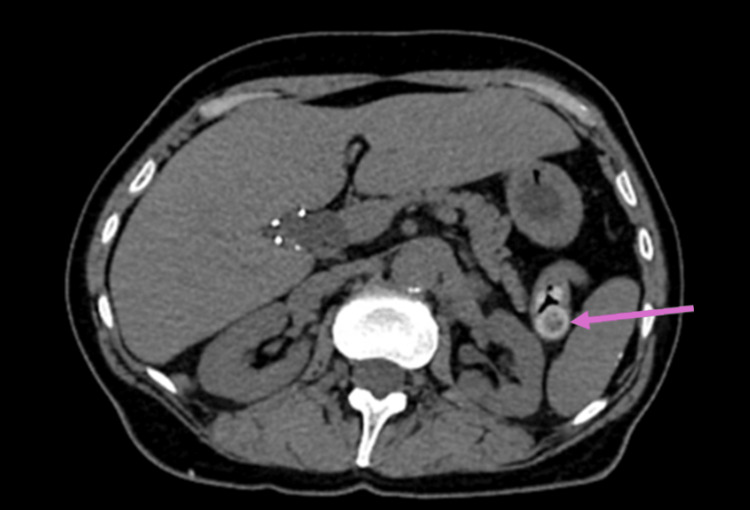
Incidental small bowel mass seen on CT angiography (pink arrow) CT: computed tomography

In addition, an incidental heterogeneous enhancing mass was seen in the small bowel in the left upper quadrant measuring 1.4×1.6 cm that was also suspicious (Figure 5). Due to this close relationship of the retroperitoneal tumor with the vasculature, a decision was also made to include vascular surgery in the planning and execution of the surgery. Small bowel resection was also recommended for the diagnosis and treatment of the mass.   

Midodrine was held preoperatively, and the patient was optimized with salt tabs and fluids for volume expansion. Prednisone was also given intraoperatively to avoid further hypotension in the perioperative period in the rare event of postoperative adrenal insufficiency. An arterial line was also placed to allow for the close monitoring of blood pressure intraoperatively.   

Through a laparotomy, the small bowel resection was performed first with quick identification and resection, followed by primary anastomosis of the jejunal mass. Attention was then diverted to the left retroperitoneal/adrenal tumor. Once the colon, pancreas, and spleen were mobilized to expose the mass, it was apparent that the mass was contiguous with the inferior-most aspect/limb of the adrenal gland. Anatomic dissection of the adrenal gland was then performed. The gland was dissected from the aorta to the level of the renal vein and circumferentially mobilized to expose the proximal and distal aspects of the renal vein in relation to the tumor. While there was no clear invasion of the mass into the vein, it was apparent that the adrenal vein had been compressed and likely occluded by the tumor, which was causing mass effect. The adrenal vein was suture-ligated at its takeoff from the renal vein and divided from the tumor. Although the tumor was difficult to separate from the renal vein, there was no obvious invasion. Therefore, careful, sharp, and blunt dissection was performed to successfully remove the tumor off the renal vein, keeping the capsule and tumor intact. The underlying renal artery was then identified and also preserved.   

The procedure proceeded as planned; however, during the exposure, manipulation, and removal of the retroperitoneal mass, the patient experienced extreme hemodynamic instability. During exposure and manipulation, acute tachycardia and hypertension upwards of 280/180 mmHg were observed. The acute hypertension and tachycardia were managed using 1 mg of phentolamine, 2.5 mg of esmolol, and a total of 900 mcg of intravenous nicardipine. In contrast, post-tumor removal, the patient experienced significant hypotension as low as 30/20 mmHg, requiring vasopressors, including 120 mcg of norepinephrine and 500 mcg of phenylephrine. Due to how labile the patient was, intravenous boluses of the antihypertensives and vasopressors were utilized rather than continuous infusions. This technique allowed the anesthesia team to maintain the goal mean arterial pressure of greater than 65 mmHg. Communication between the surgical and anesthetic team during this case was seamless and allowed for safe blood pressure management throughout.   

Emergence and extubation occurred without any incidence, and the patient was transferred to the intensive care unit without any continuous infusions. After surgery, the patient required intravenous fluid boluses, midodrine, and neosynephrine for less than 24 hours to elevate and stabilize her blood pressure. A mean arterial pressure of 60 was used for the blood pressure goal. She overall did well postoperatively and was discharged home on postoperative day 5.  

At follow-up appointments, she did have dizziness and lightheadedness; thus, her dose of midodrine was increased, and imaging was ordered. CT of the abdomen and pelvis was negative for bleeding or an acute cause of her symptoms. MRI of the brain and carotid ultrasound were also normal.  Biochemical labs were ordered to rule out adrenal insufficiency, and these were negative.   

**Figure 6:**
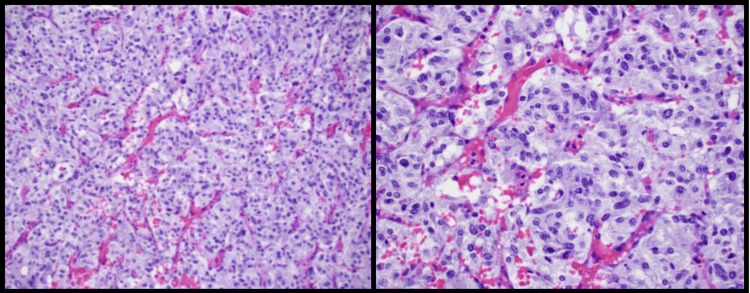
Histologic sectioning of the tumor at 4× magnification (left) and 20× magnification (right) showing neoplastic growth arising in the adrenal medulla, composed of nests of cells separated by sustentacular cells, with alternating areas demonstrating a more trabecular and solid arrangements

Final pathology confirmed the lesion to be a pheochromocytoma of low malignant potential. Histologic sectioning showed neoplastic growth arising in the adrenal medulla, composed of nests of cells separated by sustentacular cells, with alternating areas demonstrating a more trabecular and solid arrangement (Figure 6). Sections showed apparent encapsulation without definitive invasion, absence of necrosis, absence of vascular invasion, and absence of conspicuous mitosis, in an overall background of increased cellularity, and hyperchromatic cells with moderate nuclear pleomorphism. The proliferative index was low, as reflected by the Ki-67 value of less than 1%.  The final pathology was confirmed to be a 3 cm pheochromocytoma with a normal adrenal cortex, completely resected with negative margins. Per the Pheochromocytoma of the Adrenal Gland Scaled Score (PASS), this was thought to be of low malignant potential as the score was less than 4 [7]. The small bowel mass was found to be a low-grade, 1.6 cm gastrointestinal stromal tumor with 1 mitoses/5 mm^2^, also resected with negative margins.   

The patient was referred to a geneticist, and testing was negative for any mutation, including von Hippel-Lindau, multiple endocrine neoplasia, and neurofibromatosis, indicating this was likely a sporadic case. She remains on her midodrine. Repeat metanephrines and normetanephrines (plasma and urine) three and six months later were normal, and she remains free of disease.   

## Discussion

This case highlights the diagnostic complexity and treatment challenges faced in this patient with a pheochromocytoma. The first challenge in this patient's care was confirmation of a hyperfunctioning tumor. Pheochromocytomas can mimic many other medical diseases, so clarification of diagnosis and appropriate treatment was important, especially as resection may have entailed the removal of a portion of the renal vein. Rare reports of hypotension with a diagnosis of pheochromocytoma have been reported [8,9]. While the common triad of symptoms of a pheochromocytoma includes headaches, palpitations, and sweating, hypertension and/or fluctuations in blood pressure more often occur [1]. Our patient's hypotension was persistent. However, there has also been a case report of a primarily epinephrine-secreting tumor in a patient presenting with hypotension/shock [10]. A cardiac workup was performed, not revealing any other cause. 

Because her hypotension was actively treated with midodrine, this originally created a diagnostic challenge with her labs. Her metanephrines were overall normal; however, her normetanephrines were elevated. Since falsely elevated plasma metanephrines have been reported in patients taking midodrine, but not normetanephrines, a decision was made to still repeat her labs off midodrine and also obtain a 24-hour urine test to assure accuracy [6]. Repeat labs showed consistently elevated normetanephrines in the serum and urine, consistent with the findings from Emms et al. [6] that normetanephrines are not affected by midodrine. Biopsy was not recommended given the location and possible hyperfunctional characteristics of the tumor to avoid risks of hypertensive crisis, hematoma, increased risk of surgery, arrhythmia, stroke, or death [11,12]. Given the concern for a hyperfunctioning tumor, resection was planned as per the American Association of Endocrine Surgeons guidelines [5,12]. 

The second diagnostic challenge was imaging characteristics and location. While imaging showed the tumor had been present for years prior, it was slowly increasing in size. Its location made the differentiation of an adrenal tumor versus a paraganglioma difficult. The 2017 WHO classification defines pheochromocytomas as within the adrenal and paragangliomas as extra-adrenal, only distinguishing them by location [3]. However, it was unclear if this tumor was peripherally located in the adrenal or at an extra-adrenal location. Additionally, most paragangliomas may be nonfunctional [13]. However, since paragangliomas tend to produce norepinephrine and dopamine while pheochromocytomas produce epinephrine, her normal metanephrines and elevated normetanephrines suggest the tumor may have been a paraganglioma rather than a pheochromocytoma [2,14]. However, according to the WHO guidelines, the location within the periphery of the adrenal gland suggests the tumor is adrenal in origin [3]. 

Pheochromocytomas are associated with certain familial syndromes, such as von Hippel-Lindau disease, neurofibromatosis type 1, and multiple endocrine neoplasia type 2, though sporadic cases are more common [15]. For paragangliomas, somatic mutations in the HIF2A gene for polycythemia-paraganglioma-somatostatinoma syndrome should also be evaluated [16]. For this reason, genetic testing should also be performed. Assuring the lesion is unilateral and without metastatic disease is also important, especially when genetic mutations are found. Our patient was found to have a sporadic and unilateral lesion. 

This case also highlights the need to recognize and prepare for possible hemodynamic instability in both the perioperative period and intraoperatively, if pheochromocytoma is possible. Successful treatment of unrecognized pheochromocytomas is reported within the current literature, but poor outcomes can result with a lack of preparation, especially when repeated hypotensive episodes are encountered [17]. The American Association of Endocrine Surgeons Guidelines for Adrenalectomy recommends selective or nonselective blockade to safely prepare patients for surgical resection [12]. While alpha blockade is utilized to stabilize blood pressure, our patient had persistent hypotension, where there was concern that it would worsen with the initiation of alpha blockade. Perioperative hypotension and overall hemodynamic instability with the removal of any pheochromocytoma are possible and can put a patient at an increased risk for morbidity and increased intensive care unit/hospital stay [17]. Therefore, the patient was medically optimized with salt tabs and volume expansion, and coordination with anesthesia and critical care allowed the appropriate medications to be available for the rapid treatment of profound hyper- or hypotension during and after the surgery.  This multidisciplinary coordination allowed for the appropriate preparation of the patient and staff for hemodynamic variations that may have otherwise been threatening to the patient's outcome.  

## Conclusions

Lab findings and imaging were suspicious for pheochromocytoma or even paraganglioma, although they were not definitive, given the patient's clinical presentation of longstanding hypotension and location of the tumor in the renal hilum. This diagnosis was then confirmed with the final surgical pathology. This case highlights the importance of recognizing and understanding the need for multidisciplinary care to successfully treat patients, even when the clinical picture is uncertain. While a diagnosis was obtained by pathology, this case also reinforces the need for biochemical workup of adrenal and even periadrenal lesions to rule out a pheochromocytoma or functioning paraganglioma, even in the absence of the classical symptom presentation.  
